# Endometrial Patterns Among Rural Women Undergoing Gynecological Procedures in a Tertiary Care Hospital

**DOI:** 10.7759/cureus.35893

**Published:** 2023-03-08

**Authors:** Saravanakumari Vijayakumar

**Affiliations:** 1 Department of Pathology, Sri Lakshmi Narayana Institute of Medical Sciences, Pondicherry, IND

**Keywords:** histopathology, abnormal uterine bleeding, rural women, gynecological complaints, endometrial patterns

## Abstract

Introduction

Women with endometrial diseases often present in outpatient departments, and outpatient procedures easily enable diagnosis. Histopathological patterns vary with age, menstrual cycle, drug use, and pathological state. Teaching hospitals are often located away from rural areas.

Materials and methods

An ambispective study was performed over 33 months in a rural medical college hospital by perusing the histopathological registers of the department. Details of history and endometrial samples (biopsy and hysterectomy) were obtained using biopsy numbers and requisition forms. Functional and organic histopathology findings were manually entered into a spreadsheet, and statistics were performed using Statistical Package for the Social Sciences (SPSS) for Windows version 22 (IBM SPSS Statistics, Armonk, NY, USA).

Results

There were 226 women, with total abdominal hysterectomies (TAH) performed in a significantly higher proportion of middle-aged women (range: 41-59 years). Of the middle-aged women, 70% presented with excessive menstrual bleeding, and a similar percentage of older women presented with uterovaginal (UV) prolapse. Hysterectomy specimens constituted most samples in this study. Proliferative endometrium was observed in 40% of patients and metaplasia (papillary syncytial, tubal, and squamous) in only 1.7%. Normal proliferative and secretory endometrium were observed in 91 (40.3%) and 41 (18.1%) patients, respectively. The presence of hyperplasia, decidualization, stromal breakdown, adenocarcinoma, adenomyosis, and endometrial polyps did not vary significantly among the three age groups (elderly, middle-aged, and young).

Conclusion

Most women in rural areas presented to the gynecologist with uterine bleeding; middle-aged women constituted most of those with gynecological complaints. Normal endometrium was observed in nearly half of the patients. Adenomyosis was the most common cause of uterine bleeding. Uterine endometrial malignancies were rare.

## Introduction

Twenty years ago, 24.1% of rural Indian women had gynecological complaints when surveyed [1]. A quarter of the participants did not seek healthcare [1]. Globally, benign and malignant endometrial disorders contribute to significant gynecological morbidity in a broad range of women [2]. Women with endometrial diseases often present in outpatient departments, and outpatient procedures easily enable diagnosis. Histopathological patterns vary with age, menstrual cycle, drug use, and pathological state. Teaching hospitals are often located away from rural areas. Here, we present our findings in women treated at a rural medical college hospital.

## Materials and methods

We aimed to determine the spectrum of endometrial patterns in different age groups of women with gynecological problems in the rural population of Pondicherry. An ambispective analysis of all gynecological lesions treated at the Sri Lakshmi Narayana Institute of Medical Sciences between April 1, 2020, and December 31, 2022, was performed. After obtaining approval from the Institutional Research Board (number IEC/C-P/13/2021), a search was conducted in the gynecology histopathological register of the Department of Pathology. Case files and slides were obtained using biopsy numbers from the requisition forms and then reviewed. Women with gynecological complaints who underwent dilatation and curettage, endometrial pipelle biopsy, and hysterectomy were included in the study. Patients who were pregnant (complicated/uncomplicated) and those with systemic disorders that cause uterine bleeding were excluded. Endometrial samples without endometrial glands were excluded from the final analysis. Information was manually extracted, and data were entered into an Excel spreadsheet (Microsoft Corporation, Redmond, WA, USA). The information included clinical history (symptoms, signs, menstrual history, duration of illness, and parity status), physical examination, vaginal examination, investigations, and pathological diagnosis. Curated data were analyzed using the Statistical Package for the Social Sciences (SPSS) for Windows version 22 (IBM SPSS Statistics, Armonk, NY, USA). Frequencies were calculated for categorical variables, and mean±standard deviation (SD) was calculated for continuous variables. Statistical significance was considered when the P value was ≤0.05.

## Results

There were 226 patients in this study with a mean age of 45.4±8.1 years; most women were middle-aged (n=140, 62.2%). Only 21 women were ≥60 years. Total abdominal hysterectomy (TAH) was performed in a significantly higher (P=0.01) proportion of middle-aged women (range: 41-59 years). In comparison, vaginal hysterectomy was performed in a substantially higher proportion of older patients (P<0.01). Of the middle-aged women, 70% presented with excessive menstrual bleeding, and a similar percentage of older women (71.4%) presented with uterovaginal (UV) prolapse. Hysterectomy specimens constituted most samples in this study (Table 1).

**Table 1:** Endometrial findings in the study population

N=226	Number (%)
Samples	
Total abdominal hysterectomy	108 (47.8)
Vaginal hysterectomy	30 (13.3)
Dilatation and curettage	86 (38.2)
Pipelle	2 (0.9)
Symptoms	
Leukorrhea	9 (4)
Excess bleeding	157 (69.5)
Histopathological findings	
Proliferative	91 (40.3)
Secretory	41 (18.1)
Metaplasia	
Papillary syncytial metaplasia	1 (0.4)
Tubal metaplasia	2 (0.9)
Squamous metaplasia	1 (0.4)
Senile cystic atrophy	15 (6.7)
Atrophic	11 (4.9)
Basal endometrium	13 (5.8)
Disordered proliferative	12 (5.3)
Shedding endometrium	4 (1.8)
Hyperplasia/endometrial intraepithelial neoplasia	12 (5.3)
Adenocarcinoma	2 (0.9)
Endometrial polyp	7 (3.1)
Stromal breakdown	11 (4.9)
Decidualization	16 (7.1)

**Figure 1:**
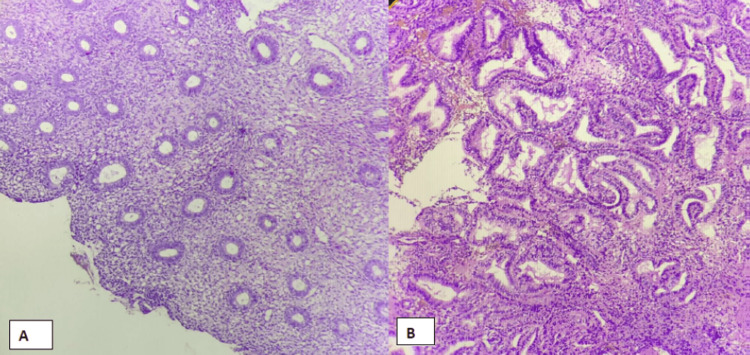
Proliferative and secretory endometrium A: Proliferative endometrium: 4×, section shows round glands lined by columnar epithelium in the background of the dense compact stroma. B: Secretory endometrium: 4×, section shows irregular convoluted glands lined by columnar to cuboidal cells with luminal secretions and variable stromal edema.

Proliferative endometrium (Figure 1) was observed in 40% of patients and metaplasia in only 1.7% (n=4). Uterovaginal prolapse and leukorrhea were not associated with metaplasia (Table 2).

**Table 2:** Comparison of symptoms with respect to endometrial findings

Pathology	Leucorrhea (number)	P-values	Excessive bleeding (number)	P-values	UV prolapse (number)	P-values
Proliferative	1	0.13	65	0.44	10	0.15
Secretory	3	0.43	34	0.06	0	0.003
Papillary syncytial metaplasia	0	<0.001	1	0.61	0	0.86
Tubal metaplasia	0		2		0	
Squamous metaplasia	0		1		0	
Senile cystic atrophy	1	0.83	0	0.001	15	0.001
Atrophy	2	0.04	2	0.001	3	0.24
Basal endometrium	1	0.76	9	0.99	2	0.97
Disordered proliferative	1	0.71	9	0.66	0	0.13
Hyperplasia/endometrial intraepithelial neoplasia	0	0.01	12	0.02	0	0.15
Adenocarcinoma	0	0.99	2	0.51	0	0.76
Endometrial polyp	0	0.90	4	0.74	2	0.31
Stromal breakdown	0	0.76	10	0.23	0	0.16
Decidualization	0	0.96	5	0.69	0	0.29
Shedding endometrium	0	0.91	4	0.80	0	0.39

**Figure 2:**
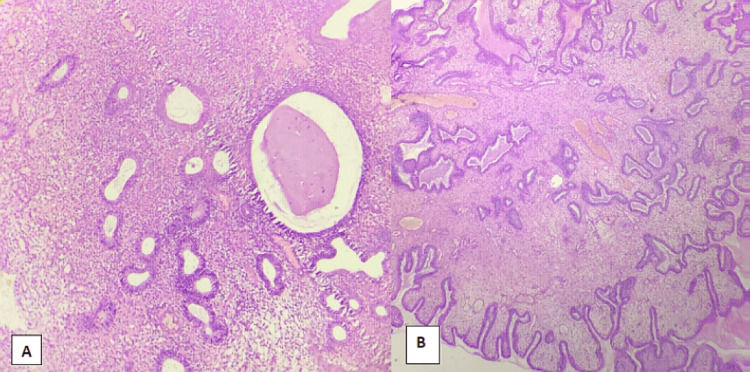
Disordered proliferative endometrium and endometrial polyp A: Disordered proliferative endometrium: 10×, section shows proliferative endometrial glands admixed with cystically dilated glands. Stroma shows variable edema and hemorrhage. B: Endometrial polyp: 4×, section shows polypoidal tissue lined by columnar epithelium with dilated glands embedded in dense fibrotic stroma admixed with thick-walled blood vessels.

**Figure 3:**
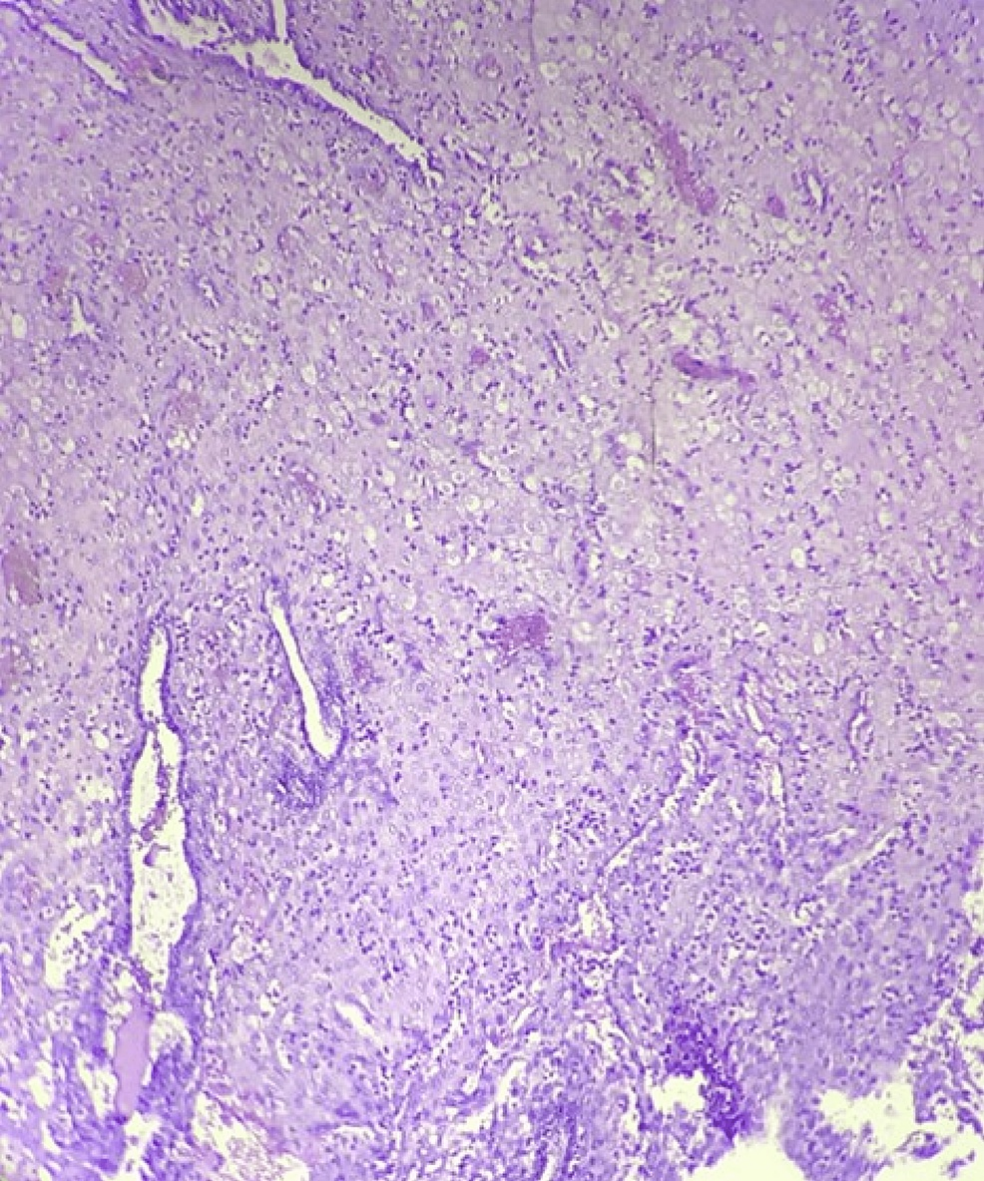
Decidualized endometrium: 10×, section shows inactive endometrial glands admixed with decidualized stroma having large cells with abundant cytoplasm and prominent cell border

Normal proliferative and secretory endometrium was observed in 91 (40.3%) and 41 (18.1%) patients, respectively. The presence of hyperplasia, decidualization, stromal breakdown, adenocarcinoma, and endometrial polyps (Figure 2 and Figure 3) did not vary significantly among the three age groups (Table 3).

**Table 3:** Age groups and endometrial histopathological findings

Findings	Elderly (≥60)	Middle (41-59)	Young (≤40)
N=226	N=64 (28.4%)	N=140 (62.2%)	N=21 (9.3%)
Proliferative	2	66	23
Secretory	0	28	13
Senile cystic atrophy	9	6	0
Atrophic	6	5	0
Basal endometrium	0	12	1
Disordered proliferative	0	8	4
Endometrial intraepithelial neoplasia/hyperplasia	0	12	0
Adenocarcinoma	1	1	0
Endometrial polyp	3	4	0
Stromal breakdown	0	6	5
Decidualization	0	12	4

## Discussion

Owing to the impact of various hormonal, dietary, and environmental factors on the endometrium, it is susceptible to multiple pathological lesions [3]. Abnormal uterine bleeding (AUB) is a pattern that does not correspond to the amount, duration, or frequency of a normal menstrual cycle [4,5]. According to reproductive function and age, the clinical presentation and mechanism of AUB and endometrial pathology vary with age [4]. The etiology of AUB may vary: young women of reproductive age present with hormonal imbalances, while perimenopausal and postmenopausal women may present with endometrial hyperplasia and neoplasia as the etiology of the bleeding [5,6]. Dysfunctional uterine bleeding is a term used to denote abnormal uterine bleeding without any organ factors, such as iatrogenic causes, reproductive tract disease, and systemic diseases [7]. Most studies have dealt only with the impact of abnormal/dysfunctional uterine bleeding and the accompanying endometrial changes [4,5]. In the current study, excessive bleeding was observed in 156 (69.3%) patients, two-thirds of this group were middle-aged women (41-59 years). Bleeding was significantly associated with atrophic endometrium, adenomyosis, and hyperplasia (Table 2). Some studies have included specimens obtained for infertility [5,8]. We did not evaluate women for infertility in this study.

Ninety-one (41.3%) specimens showed proliferative endometrium. Only Prathipaa and Divya [9] and Batra et al. [10] had similar proportions, while most other studies had percentages less than one-third of their study populations [10,11]. Less than 5% of our samples yielded atrophic endometrium, which was similar to the studies by Sajitha et al. [11], Prathipaa and Divya [9], and Forae and Aligbe [2].

There is another study on endometrial biopsies from Pondicherry, albeit from an urban area. They studied only patients with AUB, unlike the current study in which all gynecological complaints were considered. The frequencies of malignancies were similar, and the proportions of proliferative endometrium were higher (53.85% versus 40.3%), while that of secretory endometrium was similar (18.8% versus 18.1%). The frequencies of endometrial polyps and disordered proliferative endometria were similar between groups. The number of adenomyosis and metaplasia was not known in that study [12].

Endometrial polyps are a benign localized overgrowth of endometrial tissue protruding into the uterine cavity, the pathogenesis of which is unknown; however, they are believed to develop due to unbalanced estrogens and progestin [13]. Endometrial polyps were noted in <10% of the study population and were similar to the findings in studies from both the Indian subcontinent and Africa [2,11]. Simple hyperplasia was seen in <5% of patients, as in the studies by Panchal et al. [14] and Dwivedi et al. [15]. The burden of adenocarcinoma is low, with most studies reporting <10 cases. Metaplasia was not reported in any of the studies involving endometrial samples; only four patients in this study had metaplasia. All four presented with excessive menstrual bleeding.

Disordered proliferative endometrium is an exaggerated normal proliferative phase with no significant increase in gland-to-stromal ratio due to the continuous estrogen stimulus, mostly seen in perimenopausal women [10]. In the disordered proliferative endometrium, glands are irregularly dilated with focal branching and outpouching. In our study, disordered proliferative endometrium was observed in 5.3% of patients, which was similar to that of most studies, except for that of Sajitha et al. [11] and Batra et al. [10], in which >10% of their samples have disordered proliferative endometrium.

In the proliferative spectrum of lesions, one end is disordered proliferative endometrium, and endometrial carcinoma is at the other end, while endometrial hyperplasia lies in between [7]. Disordered proliferative endometrium was observed in 12 patients and adenocarcinoma in two. The risk factors of endometrial hyperplasia are a sedentary lifestyle, obesity, increased intake of animal fat, and diabetes [7]. We did not have anthropometric and biochemical data on these patients. Mostly, atypia and neoplasia are seen among postmenopausal and perimenopausal women [14]. Another common lesion in perimenopausal women is endometrial hyperplasia due to the stimulation of unopposed estrogen that may lead to hyperplasia and proliferation of the endometrium [4]. In the current study, 10/11 cases of hyperplasia were observed in middle-aged women. Women with obesity may have increased peripheral availability of estrogen due to low sex hormone-binding globulin and androgenic aromatization to estrogen in adipose tissue. Estrogenic unopposed stimulation can lead to endometrial intraepithelial neoplasia (EIN) [4]. There was only one case of EIN in our study. In EIN/atypical hyperplasia, the increased gland-to-stromal ratio with loss of polarity and pleomorphic enlarged rounded nuclei with nucleoli are seen [4]. Atypical hyperplasia will show hyperplastic endometrial glands with nuclear atypia and abundant eosinophilic cytoplasm [15]. Nuclear features are subtle and include loss of polarity, nuclear stratification, and prominent nucleoli with nuclear rounding [15].

In 1994, the WHO classification stated that endometrial hyperplasia is classified into simple and complex forms based on glandular architecture [16]. In 2014, the WHO simplified the classification into non-atypical endometrial hyperplasia (benign hyperplasia) and atypical hyperplasia/endometrial intraepithelial neoplasia/well-differentiated carcinoma [17]. In the current study, adenocarcinoma was observed in 46-year-old and 60-year-old women. The risk of atypical hyperplasia for developing endometrioid adenocarcinoma is 25%-40% [16].

In postmenopausal women, bleeding is mostly due to atrophic endometrium [11]; in our study, only four women ≥60 years presented with bleeding. The glandular architecture of the atrophic endometrium may be budded or cystic [4]. The glands are lined by mitotically inactive bland cuboidal to flattened endometrium, which is embedded in the spindled inactive stroma [4]. Thin-walled blood vessels over the expanded cystic glands are vulnerable to injury, which may be the reason for bleeding in the atrophic endometrium [4,11]. Only 2/11 patients with atrophic endometrium presented with bleeding.

Hormonal preparations that have both estrogen and progesterone will show a poor or weak secretory endometrium [16]. Progestogen-only compounds can lead to a weak secretory or atrophic endometrium stroma being expanded with pseudo-decidualization associated with mononuclear infiltrates [4].

Limitations

The study sample was small and partly retrospective, and hence, some data could not be obtained for patients, especially data related to anthropometry, comorbid metabolic diseases, biochemistry, and hematology. The study was performed in a rural medical college hospital with the nearest city eight kilometers away. Hence, the findings applicable to this rural populace may not be generalizable to other rural areas.

## Conclusions

Most women in rural areas presented to the gynecologist with uterine bleeding, and middle-aged women constituted the majority of those with gynecological complaints. Hysterectomy samples constituted the bulk of histopathological samples. Proliferative endometrium was observed in nearly half of the patients. Uterine endometrial malignancies were rare.
